# Designing a 0D/1D S-Scheme Heterojunction of Cadmium Selenide and Polymeric Carbon Nitride for Photocatalytic Water Splitting and Carbon Dioxide Reduction

**DOI:** 10.3390/molecules27196286

**Published:** 2022-09-23

**Authors:** Yayun Wang, Haotian Wang, Yuke Li, Mingwen Zhang, Yun Zheng

**Affiliations:** 1 Xiamen Key Laboratory of Optoelectronic Materials and Advanced Manufacturing, College of Materials Science and Engineering, Huaqiao University, Xiamen 361021, China; 2 Department of Chemistry and Centre for Scientific Modeling and Computation, Chinese University of Hong Kong, Shatin, Hong Kong, China; 3 Fujian Provincial Key Lab of Coastal Basin Environment, School of Materials and Environment Engineering, Fujian Polytechnic Normal University, Fuzhou 350300, China

**Keywords:** photocatalysis, water splitting, CO_2_ reduction, S-scheme heterojunction, carbon nitride, quantum dot

## Abstract

Constructing photocatalysts to promote hydrogen evolution and carbon dioxide photoreduction into solar fuels is of vital importance. The design and establishment of an S-scheme heterojunction system is one of the most feasible approaches to facilitate the separation and transfer of photogenerated charge carriers and obtain powerful photoredox capabilities for boosting photocatalytic performance. Herein, a zero-dimensional/one-dimensional S-scheme heterojunction composed of CdSe quantum dots and polymeric carbon nitride nanorods (CdSe/CN) is created and constructed via a linker-assisted hybridization approach. The CdSe/CN composites exhibit superior photocatalytic activity in water splitting and promoted carbon dioxide conversion performance compared with CN nanorods and CdSe quantum dots. The best efficiency in photocatalytic water splitting (10.2% apparent quantum yield at 420 nm irradiation, 20.1 mmol g^−1^ h^−1^ hydrogen evolution rate) and CO_2_ reduction (0.77 mmol g^−1^ h^−1^ CO production rate) was achieved by 5%CdSe/CN composites. The significantly improved photocatalytic reactivity of CdSe/CN composites primarily originates from the emergence of an internal electric field in the zero-dimensional/one-dimensional S-scheme heterojunction, which could greatly improve the photoinduced charge-carrier separation. This work underlines the possibility of employing polymeric carbon nitride nanostructures as appropriate platforms to establish highly active S-scheme heterojunction photocatalysts for solar fuel production.

## 1. Introduction

During past 50 years, the level of carbon dioxide (CO_2_) in the atmosphere has increased significantly as a result of excessive combustion of fuel [1,2]. The development of photocatalytic technology to reduce water to hydrogen (H_2_) and recycle CO_2_ into value-added hydrocarbons will help decrease the level of CO_2_ in the atmosphere and partially meet future energy requirements [3,4,5]. However, the photocatalytic efficiencies of most unitary photocatalysts can hardly meet the practical requirements primarily ascribed to the high electron-hole recombination rate. Designing efficient heterojunction photocatalysts with the boosted separation of photoinduced electron-holes remains as a great challenge in this field [6,7,8].

Polymeric carbon nitride (CN) materials have been shown to act as promising photocatalysts for multifunctional photoredox reactions such as water decomposition, CO_2_ conversion, selective organic transformation, pollutant removal, nitrogen fixation, and bacterial inactivation [9,10,11,12,13,14,15,16]. Wang et al. synthesized one-dimensional polymeric carbon nitride nanorods by using chiral mesoporous silica nanorods as a hard-template, and showed that CN nanorods exhibited stronger photocatalytic reactivity than bulk CN in water splitting and CO_2_ conversion [17]. However, the light-harvesting ability and photocatalytic activity of pristine CN and its related nanostructures remains limited [18,19,20,21,22,23,24]. The photocatalytic reactivity of CN can be further enhanced by heterostructure design to accelerate charge-carrier separation and optimize the visible light harvesting capability [25,26,27]. So far, CN have been hybridized with different metals or semiconductors to construct heterojunction photocatalysts for pollutant removal, CO_2_ conversion, and water splitting [28,29,30,31,32]. Although better charge separation has been achieved in these heterojunction systems, most previously reported heterojunction systems are based on Schottky and type II heterojunctions at the expense of photogenerated electron reduction power.

Semiconductor quantum dots (QDs) have stimulated widespread research interest in photocatalysis, which can be attributed to the unique properties of quantum size effect and multiexciton generation effect [33,34,35,36]. In particular, CdSe quantum dots (CdSe QDs) have stimulated considerable interest in photocatalytic H_2_ production due to the high surface volume ratio, size-dependent light absorption capability, and the ability to induce multiple electron and hole production via single photon absorption [37,38,39,40]. Nevertheless, the agglomeration and photocorrison issues of CdSe QDs result in decreased surface area and a stronger recombination rate of photogenerated electrons and holes.

An S-scheme heterojunction, which is composed of two n-type semiconductors with the “S” shape transfer path of photogenerated charge carriers at the interface, has been reported to possess the highest redox capacity of heterojunction with boosted photocatalytic activity for photoredox reactions [41,42,43,44,45,46]. However, there have been few reports on the construction of zero-dimensional/one-dimensional (0D/1D) S-scheme heterojunctions for photocatalytic water splitting and CO_2_ reduction. The different work function of CdSe QDs and CN nanorods is highly likely to form S-scheme heterojunctions with accelerated charge-carrier separation efficiency and promote redox activity for photoredox reactions. Furthermore, in the 0D/1D heteronanostructure, CN nanorods possess small nanoparticles and nanosheets with abundant voids and rough surfaces, and tend to form loose networks via randomly stacking, which provides an ideal host for immobilizing CdSe QDs, offers abundant active sites, and promotes the adsorption, desorption, and transportation of reactants and products.

In this paper, we describe a 0D/1D S-scheme heterojunction photocatalyst constructed by electrostatic self-assembly of CN nanorods and CdSe QD to promote water splitting and CO_2_ reduction. Both experimental studies and density functional theory (DFT) calculations confirmed the existence of an internal electric field (IEF) in the CdSe/CN heterojunction, which can more efficiently separate photoinduced charge carriers and result in stronger redox ability. The S-scheme CdSe/CN heterojunctions showed excellent activity in water splitting and reducing CO_2_ to solar fuel. This study provides a view of CN-based photocatalysts for efficient water splitting and CO_2_ photoreduction following the S-scheme electron transfer pathways.

## 2. Results and Discussion

### 2.1. Preparation of Photocatalysts

**Figure 1:**
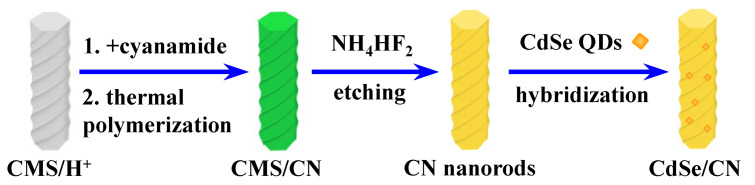
Schematic illustration of the synthetic process of CdSe/CN hybrids.

The synthetic process of CdSe/CN hybrids is shown in Figure 1. A nanocasting method was utilized to fabricate CN nanorods by using chiral mesoporous silica hard-template, and then use a linker-assisted hybridization approach to prepare CdSe QDs-modified CN nanorods. Since water-soluble CdSe QDs were covered by mercaptoacetic acid, sulfhydryl groups (-SH) and carboxylic groups (-COOH) were conjugated on the surface of CdSe QDs and ionized in water, respectively. The amino groups (-NH_2_, =NH) on the surface of CN nanorods shows a strong affinity for carboxylic acid groups (-COOH) of CdSe QDs, forming the resultant CdSe/CN hybrid materials.

### 2.2. Morphological Characterization

**Figure 2:**
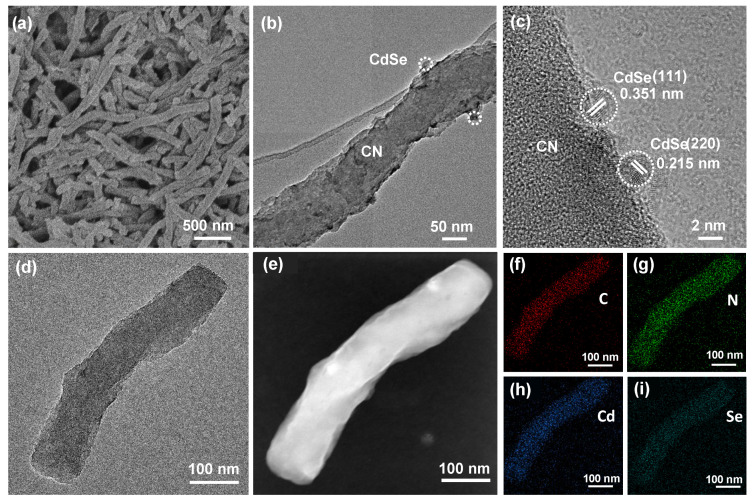
(**a**) SEM, (**b**–**d**) TEM and HRTEM images, (**e**) HAADF-STEM images, and TEM element mapping images of (**f**) C, (**g**) N, (**h**) Cd, and (**i**) Se of 5% CdSe/CN.

The morphology and nanostructure of CdSe QDs, CN nanorods, and 5% CdSe/CN were studied by scanning emission microscopy (SEM) and transmission electron microscopy (TEM). The SEM images of CN nanorods and 5% CdSe/CN both showed a uniform rod-like morphology with an outer diameter of ca. 0.15 µm and a length of ca. 2 µm (Figure 2a, Figures S1 and S2). The TEM and HRTEM images of 5% CdSe/CN presents that small nanoparticles with the size of ca. 5 nm are attached onto the surface of nanorods, confirming the formation of 0D/1D heteronanostructure (Figure 2b,c and Figure S3). The lattice spacings of the CdSe QDs were 0.215 and 0.351 nm, ascribed to (220) and (111) faces of cubic CdSe (JCPDS19-0191), respectively (Figure 2d). The high-angle annular dark-field scanning transmission electron microscopy (HAADF-STEM) images, elemental mapping images, and energy dispersive X-ray (EDX) spectrum validated the existence of C, N, Cd, and Se elements for 5% CdSe/CN composite (Figure 2e–i and Figure S4).

### 2.3. Structural Characterization

**Figure 3:**
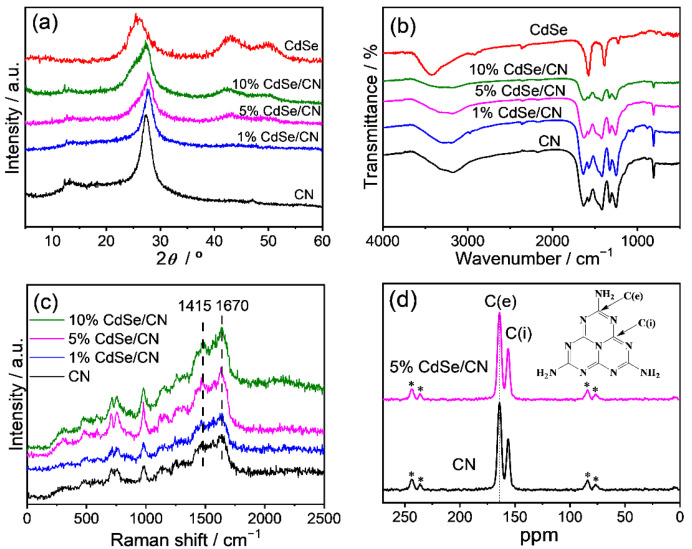
(**a**) XRD patterns, (**b**) FTIR spectra, (**c**) UV–Raman spectra, and (**d**) solid-state ^13^C NMR spectra of CdSe/CN composite and CN nanorods. The stars (*) correspond to the spinning sidebands.

The X-ray diffraction (XRD) patterns of CdSe/CN, CN nanorods, and CdSe QDs samples are demonstrated in Figure 3a. Concerning the XRD pattern of CN nanorods, the two diffraction peaks at 13.0° and 27.4° are indexed to be the (100) reflection of the continuous heptazine framework with an in-plane repetition period of 0.685 nm and the (002) reflection of the graphitic structure with d value 0.326 nm, respectively [47]. CdSe QDs have a face-centered cubic CdSe crystal structure (JCPDS19-0191). The diffraction peaks at 25.4°, 42.0°, and 49.7° correspond to the (111), (220), and (311) crystal planes of CdSe QDs. The XRD patterns of CdSe/CN hybrids exhibit diffraction peaks corresponding to both CN nanorods and CdSe QDs, indicating the presence of two phases. As the amount of CdSe QDs increases, the diffraction peaks of the CdSe/CN composites at 13.0° decrease gradually, while the diffraction peaks at 42.0° and 49.7° became increasingly more obvious. These results prove that CdSe QDs were indeed incorporated with CN nanorods.

The Fourier transform infrared (FTIR) spectra of pristine CN nanorods, CdSe QDs, and CdSe/CN hybrids are shown in Figure 3b. For CN nanorods, the stretching mode of the carbon and nitrogen heterocycle and breathing mode of the *s*-triazine unit are presented as the characteristic band in the regions of 1200–1600 and 810 cm^−1^, respectively. FTIR spectra of mercaptoacetic acid-coated CdSe QDs showed characteristic peaks at 1220, 1390, and 1580 cm^−1^, corresponding to the vibrations of hydroxyl and carboxyl groups because the ligands are attached to the nanoparticles. Characteristic bands of CN nanorods and CdSe QDs both appear in the FTIR spectra of CdSe/CN composites, confirming the emergence of composite photocatalysts. Additionally, the broadband in the region of 3000–3800 and 2349 cm^−1^ are assigned to the absorption of H_2_O and CO_2_ on the catalysts from the atmosphere.

The Raman spectra were acquired to investigate the chemical structure of CdSe/CN (Figure 3c). There are not any bands (in the region of 2000–2500 cm^−1^) assigned to triple C≡N units or N=C=N groups of CN structure. The bands in the range 1200–1700, 690, and 980 cm^−1^ are assigned to the C-N tensile vibration of disordered graphitic carbon-based materials, double degenerate mode of bending vibration in the plane of heptazine, and the symmetric N-breathing mode of the heptazine unit, respectively. The peaks at ca. 1415 and 1620 cm^−1^ are assigned to the D (disorder) and G (graphitic) bands of CN, related to structurally disordered graphitic carbons and other materials containing layered carbon and nitrogen. These features were observed for all CN nanorods and CdSe/CN composite catalysts.

Additionally, solid-state ^13^C NMR spectra showed that the heptazine units were presented for both CN nanorods and 5% CdSe/CN (Figure 3d). The peaks at ca. 164.3 and 155.6 ppm correspond to the C (e) atom of [CN_2_(NH_x_)] and C(i) atoms of melem (CN_3_) of poly (heptazine) structures. Raman spectra and ^13^C NMR spectra showed that there was a graphitic structure comprising heptazine heterocycles in the CdSe/CN composites.

**Figure 4:**
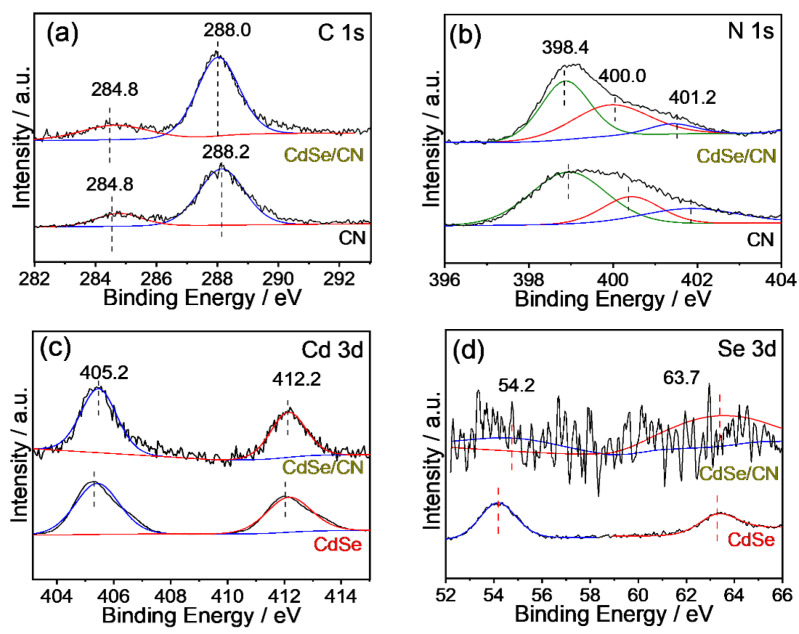
XPS spectra of 5% CdSe/CN: (**a**) C 1s, (**b**) N 1s, (**c**) Cd 3d, and (**d**) Se 3d.

The chemical state of the CdSe/CN hybrid was measured by X-photoelectron spectroscopy (XPS). Six elements (C, N, Cd, Se, O, and S) were determined for the XPS survey spectra of 5% CdSe/CN (Figure S5). In comparison with CdSe, an additional N peak at the shoulder next to the Cd peaks is observed for CdSe/CN hybrid, which confirms the presence of additional CN in the composite. Apart from Cd and Se, other elements of C, O, and S for CdSe/CN hybrid originate from the mercaptoacetic acid ligand that encapsulated the CdSe QDs. The two peaks centered at 284.8 and 288.3 eV for the C1s spectrum belong to sp^2^ C-C and sp^2^-hybridized carbon in the N aromatic ring (N-C=N), respectively (Figure 4). Three peaks centered at 398.5, 400.0, and 401.2 eV for the N 1s spectrum are ascribed to be the sp^2^-hybridized nitrogen in the triazine ring (C-N=C), the tertiary nitrogen N-(C)_3_ group, and amino functions (C-N-H) due to incomplete polymerization of poly (tri-s-triazine) structures. The sp^2^-hybridized nitrogen in the triazine ring (C-N=C, 398.5 eV), the tertiary nitrogen group (N-(C)_3_, 400.0 eV), and sp^2^ hybrid carbon (N-C=N, 288.0eV) comprise heptazine heterocyclic ring units of CN polymers. The two peaks at 405.0 and 412.0 eV for the Cd 3d spectrum correspond to Cd 3d_5/2_ and Cd 3d_3/2_, respectively. The two peaks at 54.0 and 63.5 eV are assigned to Se 3d and selenium oxide (formed by the partial oxidation of CdSe QDs in the air), respectively. Based on the XPS spectra of CN nanorods, CdSe QDs, and 5% CdSe/CN, it can be concluded that CdSe QDs are successfully hybridized with CN nanorods. In particular, the binding energies of C 1s and N 1s of 5% CdSe/CN were shifted negatively by 0.2 eV compared with the original CN, and binding energy Cd 3d and Se 3d of 5% CdSe/CN was more positive compared to the pristine CN, implying the existence of charge transfer pathways between CdSe QDs and CN nanorods. 

### 2.4. Photochemical Properties and Band Structure

**Figure 5:**
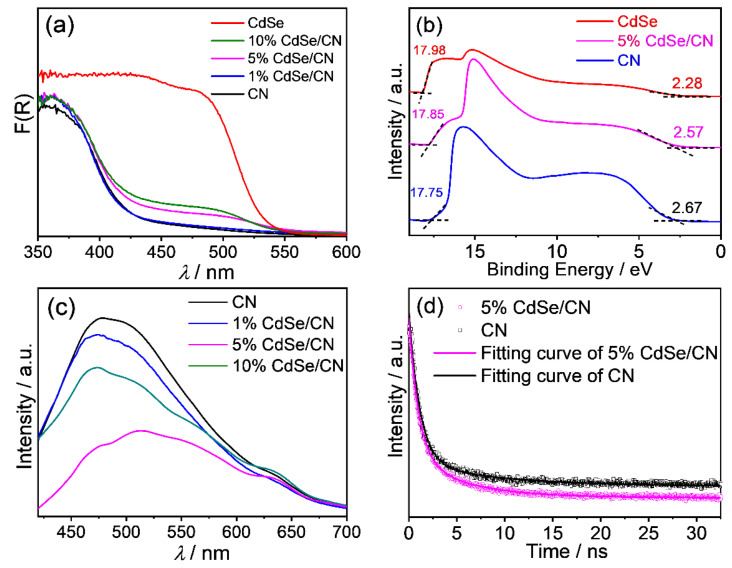
Optical properties of CdSe/CN: (**a**) UV–vis DRS spectra; (**b**) UPS valence band (VB) spectra of CN, 5% CdSe/CN, and CdSe; (**c**) steady-state photoluminescence spectra; and (**d**) time-resolved photoluminescence spectra of CdSe/CN composites at room temperature.

The electronic properties and light-harvesting ability were explored via UV–vis diffuse reflectance spectroscopy (DRS). The CdSe QDs exhibits obvious visible-light absorption with a band edge of 521 nm (Figure 5a). The pristine CN nanorods sample presents its basic absorption edge at 452 nm. All CdSe/CN hybrids exhibit stronger visible-light absorption ability than pristine CN nanorods. As the CdSe QDs content increases, the coverage spectrum of the composite sample becomes wider and the color of the sample becomes redder. Based on the Tauc plots, the bandgap values of CN nanorods, 5% CdSe/CN, and CdSe QDs are 2.74, 2.67, and 2.38 eV, respectively (Figure S6). 

The work function (*Φ*) and valance band potential (*E*_VB_) of CN, CdSe/CN, and CdSe were monitored by ultraviolet photoelectron spectroscopy (UPS) measurement (Figure 5b). The work function (*Φ*) is ascertained by the difference between the photon energy (21.22 eV) and the binding energy of the secondary cutoff edge. The secondary cutoff edge values of CN, CdSe/CN, and CdSe were 17.75, 17.85, and 17.98 eV, respectively. The work function of CN, CdSe/CN, and CdSe were 3.47, 3.37, and 3.24 eV vs. vacuum level, respectively. Thus, the Fermi energy level of CN, CdSe/CN, and CdSe are determined to be −0.97, −1.07, and −1.20 V vs. reversible hydrogen electrode (RHE). 

The UPS widths (∆*E*) of CN, CdSe/CN, and CdSe are 15.11, 15.28, and 15.70 eV, respectively. The *E*_VB_ of the catalysts are determined according to Equation (1):

*E*_VB_ = ∆*E* − 21.22 eV (1)

The *E*_VB_ values of CN, CdSe/CN, and CdSe are estimated to be 6.11, 5.94, and 5.52 eV (vs. vacuum level). Since the reference standard 0 V vs. RHE (reversible hydrogen electrode) equals to −4.44 eV vs. vacuum level, the calculated value in eV is converted to potentials in volts. The *E*_VB_ values of CN, CdSe/CN, and CdSe correspond to 1.67, 1.50, and 1.08 V vs. RHE, respectively.

Based on the *E*_VB_ and *E*_g_ of the photocatalyst, the conduction band potential (*E*_CB_) of the photocatalysts are calculated based on Equation (2):

*E*_CB_ = *E*_VB_ − *E*_g_ (2)

Thus, the *E*_CB_ of CN, 5% CdSe/CN, and CdSe QDs are −1.07, −1.17, and −1.30 V vs. RHE, respectively.

The photoluminescence (PL) spectra of the CdSe/CN hybrids were tested with light excitation of 400 nm (Figure 5c). The primary emission band of CN nanorods is centered at ca. 480 nm. The photoluminescence intensity of CN nanorods is the largest among these samples, indicating that the original CN nanorods have the highest exciton energy and electron-hole recombination rate among these samples. This energy-wasteful process can be greatly suppressed by constructing an ideal heterostructure system on the surface of CN nanorods via integration with CdSe QDs. The photoluminescence intensity of CdSe/CN samples is remarkably reduced in comparison with that of CN nanorods. As the CdSe QDs content rises, the photoluminescence intensity of the CdSe/CN composite gradually decreases. Coating CN nanorods with CdSe QDs restricts the recombination of photoinduced charge carriers. 

The transient PL spectra of CN and CdSe/CN are shown in Figure 5d. The short lifetime (***τ***_1_) reflects radiative processes such as the recombination of the photogenerated charge carriers resulting in fluorescent emission, and the long lifetime (***τ***_2_) reveals nonradiative energy transfer processes. The short lifetimes (***τ***_1_) are 1.2 and 1.0 ns for CN and CdSe/CN, at 86.3% and 76.9%, respectively. Their radiative lifetimes are similar, but the percentage of photogenerated charge carriers on CdSe/CN is significantly reduced. This result implies that the recombination rate of photoinduced electron-hole pairs on the CdSe/CN composite are effectively suppressed after incorporating CdSe QDs with CN nanorods. Correspondingly, the nonradiative lifetimes (***τ***_2_) of CN and CdSe/CN composites are 9.7 and 5.8 ns, at 13.7% and 23.1%, respectively (Table S1). The percentage of the long lifetimes (***τ***_2_) for CdSe/CN composites is significantly higher than CN, showing a higher probability and priority of photogenerated charge carriers to participate in a series of photocatalytic reactions. The average lifetimes (***τ***_av_) of CN and CdSe/CN composite samples are further calculated to be 2.3 and 2.1 ns, respectively. These PL results indicate the formation of hybrid structures lowers charge-carrier recombination and induces efficient photoinduced charge separation for improving photocatalytic efficiency.

### 2.5. Photocatalytic Water-Splitting Activities

**Figure 6:**
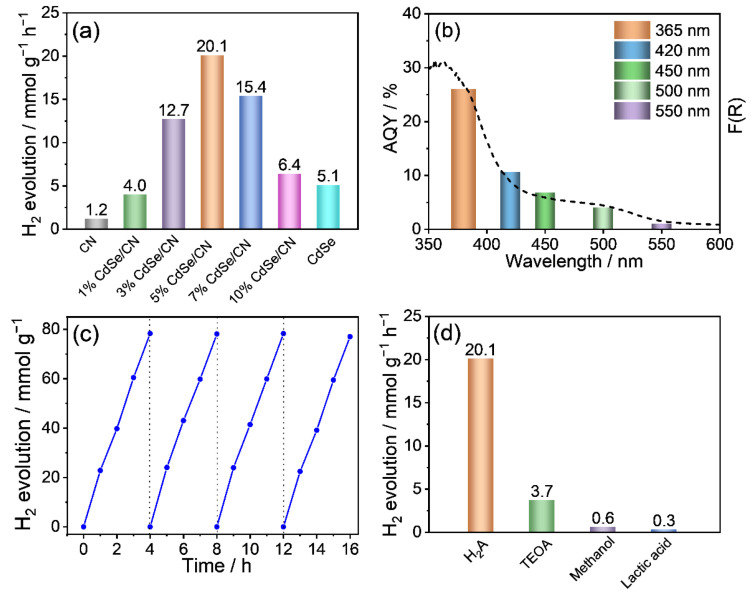
(**a**) Photocatalytic hydrogen evolution rates over CdSe/CN photocatalysts with different CdSe loading amount using ascorbic acid (H_2_A) as sacrificial reagent at pH 4.0. (**b**) Hydrogen evolution rates evolved from 5% CdSe/CN by changing sacrificial reagent. (**c**) Wavelength-dependent hydrogen evolution rates of 5% CdSe/CN. (**d**) Time-dependent photocatalytic hydrogen evolution rates over 5% CdSe/ CN.

The photocatalytic hydrogen evolution rates (HERs) of the prepared samples loaded with 3 wt% Pt using ascorbic acid (H_2_A) as the sacrificial reagent at pH 4.0 are shown in Figure 6a. The CN nanorods exhibit a low hydrogen production rate (1.2 mmol g^−1^ h^−1^). When CN nanorods are integrated with CdSe QDs, the hydrogen production rate of CdSe/CN is greatly improved. Specifically, when the weight percentage of CdSe QDs reached 10 wt%, the peak photocatalytic activity for CdSe/CN was achieved at 20.1 mmol g^−1^ h^−1^. This value is 19-fold of CN nanorods and 4-fold of bare CdSe QDs. Nonetheless, when the amount of CdSe/CN increased to 20%, the photocatalytic activity of CdSe/CN was significantly reduced. This is because the light scattering effect and shadow effect of CdSe QDs can greatly block the absorption of incident light by CN materials, and the aggregation of excessive CdSe QDs could generate the recombination center of electron-hole pairs.

As can be seen in Figure 6b, four different electron sacrificial agents including ascorbic acid (H_2_A), triethanolamine (TEOA), methanol, and lactic acid were chosen to investigate the photocatalytic hydrogen production of CdSe/CN. It is interesting to find that the rate of hydrogen production for CdSe/CN in H_2_A is obviously advantageous over the other three systems. It is worth noting that the photocatalytic H_2_ evolution activity in acidic condition (~pH 4) by ascorbic acid as the sacrificial reagent is much superior compared to the basic condition (~pH 11) by TEOA. This can be associated with the strong impact of pH value on the photocatalytic H_2_ production activity of CdSe/CN composite.

Furthermore, the effect of pH on the photocatalytic efficiency of CdSe/CN was studied at pH 2.0, 3.0, 4.0, 7.0, and 9.0 (Figure S7a). The photocatalytic H_2_ evolution rate reached its highest value, 20.1 mmol g^−1^ h^−1^, with pH 4.0. This is due to more efficient dissociation of H_2_A toward HA^−^ considering the pKa1 of H_2_A as 4.0, which provides more HA^−^ species acting as the sacrificial reductant to capture holes so that more photogenerated electrons can participate in proton reduction of hydrogen production. Moreover, the acidic reaction medium (~pH 4) can also help reduce the reduction potential of water, resulting in enhanced photocatalytic H_2_ activity.

The apparent quantum yield (AQY) of H_2_ production for 5% CdSe/CN hybrid loaded with 3 wt% Pt using ascorbic acid (H_2_A) as the sacrificial reagent at 420 nm is 10.2%, surpassing the AQYs for most of the previously reported CN-based photocatalysts (Table S2). The AQY of 5% CdSe/CN under different wavelength range coincide well with its optical absorption feature (Figure 6c), suggesting that the photocatalytic reaction is initiated by the captured photons. Next, the relationship between the H_2_ production rate and the amount of catalyst was studied (Figure S7b). With the increasing weight of catalyst, the AQY of CdSe/CN for photocatalytic hydrogen production increased first, and then reached the maximum value of 10.2% at 420 nm with the weight of 50 mg. When further increasing the weight of CdSe/CN catalyst above 50 mg, the AQY value slightly decreased and then remained stable.

The optimal 5% CdSe/CN photocatalyst was recycled for 16 h in four cycles in water-splitting arrays to explore the stability of the photocatalyst. Under light conditions, the hydrogen production rate on 5% CdSe/CN did not change significantly after four cycles of tests (Figure 6d). No noticeable changes were found in the XRD patterns, FTIR spectra, or Raman spectra of 5% CdSe/CN composite before and after photocatalytic hydrogen production, demonstrating the good stability of CdSe/CN composites (Figures S8 and S9).

### 2.6. Photocatalytic CO_2_ Reduction Activities

Photocatalytic CO_2_ reduction arrays of the samples were conducted and some reference experiments were carried out. No detectable amount of H_2_ and CO was determined without catalyst or light (Table S3). No detectable amount of CO was noticed when replacing CO_2_ with Ar gas, meaning that the decomposition of catalysts or organic additives (e.g., triethanolamine and 2,2′-bipyridyl) does not generate CO. The addition of cobalt ions (with organic ligands) cannot induce CO_2_ conversion alone. These reference experiments proved that photoreduction reactions cannot occur without any component in the photosystem (e.g., photocatalyst, Co(bpy)_3_^2+^, triethanolamine, CO_2_). Other products such as methane and methanol could be hardly generated in this photocatalytic CO_2_ reduction system, in good accordance the results of previous work [48].

**Figure 7:**
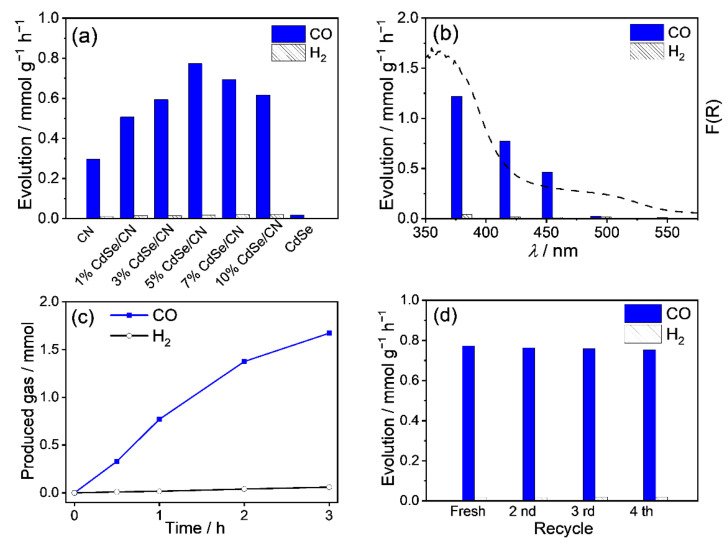
(**a**) Photocatalytic activity of CdSe/CN photocatalyst with different weight ratio in the conversion of CO_2_ to CO, and (**b**) wavelength-dependent CO and H_2_ production amount of 5% CdSe/CN composite. (**c**) Photocatalytic performance of CdSe/CN in CO_2_-to-CO conversion. (**d**) Time-dependent CO production for 5% CdSe/CN, and (**d**) stability test for 5% CdSe/CN photocatalyst in CO_2_ reduction.

All CdSe/CN showed higher CO and H_2_ yield than that of CN nanorods and CdSe. The highest CO yield of 5% CdSe/CN is 0.77 mmol g^−1^ h^−1^ with a turnover number of 23.7% and selectivity of 97.9% (Figure 7a and Table S4). The yields of CO and H_2_ decrease with increasing illumination wavelength range, suggesting the photocatalytic CO_2_ reduction is driven by the harvested photons (Figure 7b). The production amounts of CO and H_2_ tend to increase gradually in a nonlinear model with the increasing reaction time for the photocatalytic CO_2_ reduction system (Figure 7c). To test the photostability of the CdSe/CN mixture, the CO_2_ reduction reaction was performed four times. No significant loss in CO_2_ reduction activity was noticed (Figure 7d). XRD patterns, FTIR spectra, and Raman spectra of the CdSe/CN samples after photocatalytic reaction were monitored. The major chemical structure and morphology of CdSe/CN remained almost unchanged, which confirmed the stability of CdSe/CN during photocatalytic reactions (Figure S10).

### 2.7. Charge Transfer Process

**Figure 8:**
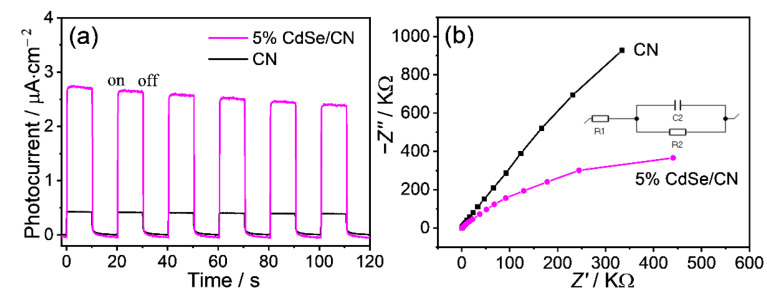
(**a**) Periodic on–off photocurrent response and (**b**) EIS Nyquist plots for 5% CdSe/CN composites and CN nanorods provided by drawing Z′ versus -Z″. Z′ and Z″ are defined by the real and imaginary part of impedance, respectively.

The photoelectrochemical capability of pristine CN nanorods and CdSe/CN composites was evaluated. Transient photocurrent responses of CdSe/CN and CN nanorods for several on–off cycles were recorded (Figure 8a). At the end of irradiation, the photocurrent value quickly decreased to zero, revealing the photoexcitation properties of the process. Five percent CdSe/CN showed nearly 5-fold enhanced photocurrent higher than pristine CN nanorods, suggesting the enhanced mobility of photoexcited charge carriers. Moreover, electrochemical impedance spectroscopy (EIS) showed a significant decrease in the diameter of 5% CdSe/CN compared to CN nanorods, suggesting that CdSe/CN composites possess boosted charge-separation efficiency (Figure 8b). The obtained semicircle can be simulated by the electrical equivalent circuit model, as shown in the inset of Figure 8b. The diameter of EIS means a charge transfer resistance at the electrode/electrolyte interface (R_2_) (Table S5). In comparison with CN, the smaller R_2_ of 5% CdSe/CN indicates decreased charge-transfer resistance, higher electrical conductivity, and accelerated migration of photogenerated charges.

Mott–Schottky experiments were also performed to explore the relative position of the conduction band (CB) edges of CN nanorods and CdSe QDs (Figure S11). Because of the positive slope, CN nanorods, 5% CdSe/CN composites, and CdSe QDs possess the feature of n-type semiconductors. The flat band potentials of CN nanorods, 5% CdSe/CN, and CdSe QDs tests resulted in −0.95, −1.07, and −1.20 V vs. RHE at pH 7, respectively. The flat band potentials are in good accordance with the results of calculated *E*_CB_ for CdSe/CN composites. 

From the slopes of the Mott–Schottky plots (Figure S12), carrier densities of CN and 5% CdSe/CN samples were calculated to be ~10^20^ and ~10^21^ cm^–3^, respectively, using the Mott–Schottky relation [49]. CdSe/CN composite showed one order of magnitude increased carrier concentration compared with pristine CN, which is beneficial for boosting the photocatalytic activity.

Linear sweep voltamentary (LSV) curves for CdSe/CN and CN are shown in Figure S13. Under current density of −10 mA cm^−2^, the overpotentials of CN and 5% CdSe/CN were found to be −210 and −150 mV, respectively. CdSe/CN presents a lower overpotential than CN, which demonstrates the construction of CdSe/CN hybrid is favorable for H_2_ production in photocatalytic H_2_ evolution.

The electronic band structure information of the CdSe/CN sample was further tested by electron paramagnetic resonance (EPR) at room temperature (Figure S14). Both CN and CdSe/CN presented one single Lorentzian line at 3515 G with a g value of 2.0034, which is ascribed to an unpaired electron on the carbon atoms of the aromatic rings within π-bonded nanosized clusters. In comparison with CN, the stronger spin intensity of CdSe/CN confirmed the promoted formation of unpaired electrons. A slightly enhanced EPR intensity under visible light illumination of CdSe/CN suggested that photochemical formation of radical pairs was promoted in the CdSe/CN semiconductor.

### 2.8. Photocatalytic Mechanism 

A further theoretical study by DFT calculation was conducted on the CdSe/CN composite heterojunction to understand the electron transfer process and the intrinsic photocatalytic mechanism.

The work functions (*Φ*) assigned to CN and CdSe were calculated according to Equation (3):

*Φ* = *E*_vac_ − *E*_F_ (3)

where *E*_F_ and *E*_vac_ represent the Fermi level and the energy of stationary electrons in a vacuum, respectively. 

**Figure 9:**
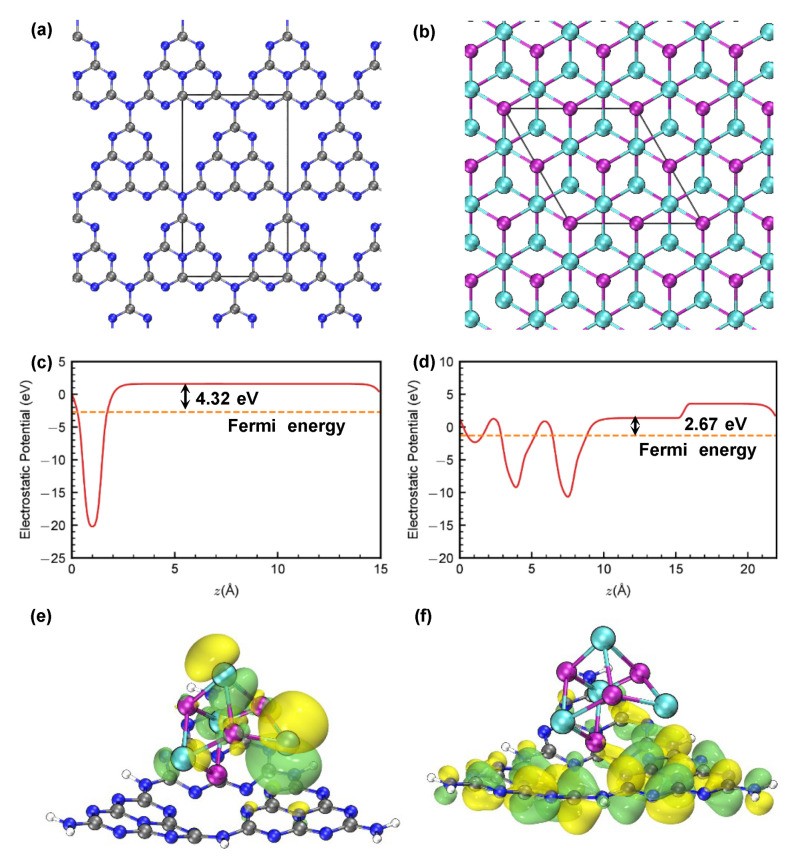
Structure of (**a**) CN and (**b**) CdSe. Electrostatic potential of (**c**) CN and (**d**) CdSe. (**e**) HOMO and (**f**) LUMO of CdSe/CN composites.

Based on DFT calculation, the work functions of CN and CdSe are 4.32 and 2.67 eV, respectively (Figure 9a–d). Since CN possessed a higher work function than that of CdSe, the electrons would transfer from CdSe to CN until the *E*_F_ level reach the same levels, and the formed IEF at the heterointerface greatly facilitates the separation of photoinduced charge carriers.

In addition, the calculation models based on CdSe/CN composites are presented in Figure 9e,f. For CdSe/CN composites, the lowest unoccupied molecular orbital (LUMO) and highest occupied molecular orbital (HOMO) are separately located at CN and CdSe, respectively. This suggests that CdSe and CN act as electron donor and acceptor, respectively; thus, the electrons could transfer from CdSe to CN.

**Figure 10:**
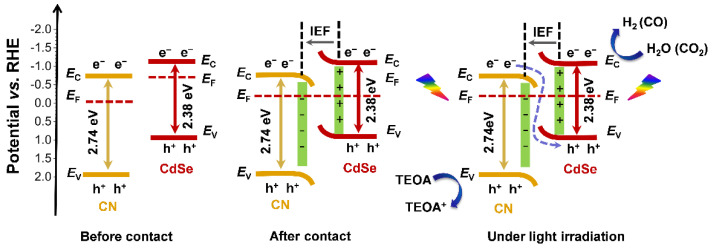
Schematic diagram of CdSe/CN composite for photocatalytic water splitting and CO_2_ reduction using triethanolamine (TEOA) as a sacrificial agent and the formation of internal electric field (IEF). The potentials are relative to reversible hydrogen electrode (RHE).

A direct S-scheme photocatalytic reaction pathway based on calculation and experimental results is illustrated in Figure 10. Since CN has a higher work function than CdSe, the photogenerated electrons transfer from CdSe to CN until their *E*_F_ levels reach the same level, and IEF is produced at the contact interface due to the different electron densities [50,51,52]. Motivated by the IEF, the photogenerated electrons in the CB of CN combine with the holes in the VB of CdSe, similar to an S-path of charge transfer [53]. The electron transfer from the CB of CdSe to the CB of CN is then prohibited. In addition, the electrons in the CB of CdSe are transferred to its surface, thus increasing the electron density of CdSe. For water splitting, the electrons on the CB of CdSe migrate to the Pt nanoparticles and then take part in the water-splitting reactions. For CO_2_ reduction, the electrons on the CB of CdSe initiate the redox reaction of the electron mediator Co(bpy)_3_^2+^, and then drive the reduction of CO_2_ to CO. The photogenerated holes are consumed by sacrificial agents such as TEOA or H_2_A. Thus, the accelerated charge separation and transfer rate is realized by constructing a 0D/1D S-scheme heterojunction of CdSe QDs and CN nanorods, thus significantly raising the photocatalytic efficiency for H_2_ evolution and CO_2_ reduction.

## 3. Materials and Methods

### 3.1. Materials

Sodium hydroxide (NaOH, ≥96.0%), ammonium bifluoride (NH_4_HF_2_, ≥98.0%), cadmium chloride hemi(pentahydrate) (CdCl_2_·2.5 H_2_O, ≥99.0%), sodium borohydride (NaBH_4_, 96%), chloroplatinic acid hexahydrate (H_2_PtCl_6_·6H_2_O, AR), cobalt(Ⅱ) chloride hexahydrate (CoCl_2_·6H_2_O, ≥99.0%), 2,2′-bipyridyl (bpy, ≥99.5%), sodium sulfide nonahydrate (Na_2_S·9H_2_O, 98.0%), mercaptoacetic acid (MPA, C_2_H_4_O_2_S, ≥90.0%), sodium sulfate (Na_2_SO_4_, ≥99.0%), selenium (Se, ≥99.0%), hydrochloric acid (HCl, 36.0~38.0%), acetone (C_3_H_6_O, ≥99.5%), ethanol (C_2_H_6_O, ≥99.5%), petroleum ether (AR), triethanolamine (denoted as TEOA, C_6_H_15_NO_3_, AR), acetonitrile (C_2_H_3_N, ≥99.8%), ascorbic acid (denoted as H_2_A, C_6_H_8_O_6_, ≥99.7%), N,N-dimethylformamide (C_3_H_7_NO, ≥99.5%) and Na_2_SO_3_ (sodium sulfite, ≥97.0%) were obtained from the China Sinopharm Chemical Reagent Co. Ltd. Tetradecanoyl chloride (CH_3_(CH_2_)_8_COCl, 97%), tetraethoxysilane (Si(OC_2_H_5_)_4_, 98%), 3-aminopropyl triethoxysilane (H_2_N(CH_2_)_3_Si(OC_2_H_5_)_3_, ≥98%), and cyanamide (NCNH_2_, 98%), D-alanine (C_3_H_7_NO_2_, ≥98%) were purchased from Sigma-Aldrich (Merck KGaA, Darmstadt, Germany). Carbon dioxide (super-grade purity, 99.999%), argon (super-grade purity, 99.999%), and nitrogen gas (99.99%) were obtained from Fujian Nanan Chenggong Gas Co. Ltd. (Fujian, China). Ultrapure water (18 mW cm^−1^) was produced by a Millipore Milli-Q water purification system (Darmstadt, Germany). All reagents were utilized without purification.

### 3.2. Synthesis of N-Myristoylalanine (C_14_-d-Ala)

C_14_-d-Ala was synthesized according to [54]. D-alanine (0.24 mol, 21.4 g) was mixed with deionized water (140 mL), NaOH (19.2 g), and acetone (120 mL). Under vigorous stirring at 0 °C, the mixture was dropwise added to tetradecanoyl chloride (0.2 mol, 49.3 g). Additionally, 20 mL 0.2 mol L^−1^ NaOH solution was injected to maintain the pH at ~12. After reaction for 1 h, a certain amount of HCl solution was added to adjust the pH at 1. The solids were washed with deionized water until neutral, cleaned with petroleum ether for several times, and vacuum dried at 50 °C. The yield of C_14_-d-Ala was 30~35 g.

### 3.3. Synthesis of Chiral Mesoporous Silica Hard-Template

Chiral mesoporous silica was synthesized according to [55,56]. C_14_-d-Ala (0.321 g, 1.0 mmol) surfactant was dissolved in water (22.1 g) and 0.01 M hydrochloric acid (10 g, 0.1 mmol), and then dropwise added to a mixture of tetraethoxysilane (1.40 g, 6.7 mmol) and 3-aminopropyl triethoxysilane (0.23 g, 1.0 mmol) with stirring at 400 rpm for 10 min. The mixture remained under static conditions at room temperature for 22 h, and then collected by filtration. The precipitates were dried at 80 °C for 12 h, and heated at 550 °C for 6 h in air. The yield of chiral mesoporous silica was 0.1~0.2 g.

### 3.4. Preparation of CN Nanorods

CN nanorods were prepared by a hard-templating method using chiral mesoporous silica nanorods as hard-templates based on [17]. Chiral mesoporous silica powder was dispersed in hydrochloric acid solution (1 mol L^−1^) at 80 °C for 20 h, centrifuged, and dried at 80 °C for 10 h. The acidified chiral mesoporous silica powder (1.0 g) was mixed with cyanamide (6.0 g) in a flask, vacuum degassed for 5 h, and sonicated at 60 °C water bath for 5 h. The mixture was washed with water, stirred for 15 min, and centrifuged. The white solids were dried at 80 °C overnight, and heated at 550 °C for 240 min at a rate of 2.2 °C·min^−1^ with the flow of nitrogen. The yellow solids were mixed with ammonium bifluoride solution (4 mol L^−1^) for 10 h, cleaned with water and ethanol 4 times, and finally vacuum dried at 80 °C for 10 h.

### 3.5. Preparation of Water-Soluble CdSe QDs

CdSe QDs were obtained based on [35,36]. In a three-necked flask, CdCl_2_·2.5 H_2_O (5 mmol, 1.142 g) was dissolved in water (60 mL) and degassed with N_2_ bubbles for 60 min. This solution was added to mercaptoacetic acid (0.85 mL), and dropwise added to sodium hydroxide (1 mol L^−1^, 24 mL) solution to tune pH value to 7. NaHSe solution was prepared by mixing Se powder (0.19 g) with sodium borohydride (0.19 g) in water (12 mL), and then injected into the above solution under high-speed stirring. The mixture was refluxed at 80 °C for 240 min, and then added to ethanol (125 mL) and centrifuged. The precipitates were totally cleaned with water and methanol, and vacuum dried at 60 °C for 10 h to obtain the CdSe QDs powder.

### 3.6. Synthesis of CdSe/CN Composites

CdSe/CN photocatalysts were prepared by a linker-assisted hybridization approach. The binding of CdSe QDs to CN nanorods can be achieved via the assistance of mercaptoacetic acid, which is a stabilizer and a bifunctional linker of CdSe QDs. Two-tenths gram of CN nanorods powder was added to 5 mL of water and a suitable amount of CdSe QDs solution (10 mg mL^−1^) and stirred at 80 °C for 12 h to acquire mixed solid by removing water. The as-prepared CdSe QDs modified CN nanorods sample was named as x% CdSe/CN, where x represents the weight percentage of the CdSe QDs to the CN nanorods (x = 1, 10, or 20). 

### 3.7. Characterizations

Scanning emission microscope (SEM) analysis was carried out via an S4800 Field Emission Scanning Electron Microscope (Hitachi, Chiyoda, Tokyo, Japan). Transmission electron microscopy (TEM) analysis was performed via a Talos F200X (Thermo, Waltham, MA, USA) and TECNAI G2F20 instrument (FEI, Hillsboro, OR, USA). X-ray diffraction (XRD) patterns were obtained from a D/MAXRB diffractometer (Rigaku, Akishima-shi, Tokyo, Japan) with Cu-Kα radiation (λ = 1.54184 Å). Fourier transform infrared (FTIR) spectra were gathered from a Nicolet iS10 FTIR spectrometer (Thermo, Waltham, MA, USA). UV–Raman scattering tests were performed with a multichannel modular triple Raman system (Renishaw Co., Wotton-under-Edge, Gloucestershire, UK) with confocal microscope at room temperature using a 325 nm laser. Solid-state ^13^C cross-polarization nuclear magnetic resonance (^13^C NMR) spectra were obtained using an Advance III 500 Spectrometer (Bruker, Billerica, MA, USA). X-ray photoelectron spectroscopy (XPS) was performed on an ESCALAB250 instrument with a monochromatized Al Kα line source (200 W) (Thermo Scientific, Waltham, MA, USA). All binding energies were referenced to the C 1s peak at 284.8 eV of surface adventitious carbon. The UV–vis diffuse reflectance spectra (DRS) were tested on a Shimadzu UV-2550 UV–vis–NIR system (Kyoto, Japan). Photoluminescence (PL) spectra were measured on a FLS-920 spectrophotometer (Edinburgh, Livingston, West Lothian, UK). Ultraviolet photoelectron spectroscopy (UPS) was performed on a PHI 5000 Versaprobe III instrument (Chigasaki, Kanagawa, Japan). UPS measurements were conducted with an unfiltered He I (21.22 eV) gas discharge lamp and a gold calibration. The width of binding energy (∆*E*) was determined from the two intersections with the UPS spectrum baseline. The width value of He I UPS spectra (21.22 eV) was used as the standard. Since the reference standard 0 V vs. RHE (reversible hydrogen electrode) equals –4.44 eV vs. vacuum level, the calculated value in eV was converted to potentials in volts. Electron paramagnetic resonance (EPR) measurements were carried out on a Bruker model A300 spectrometer (Billerica, MA, USA).

### 3.8. Photoelectrochemical Measurement

To prepare the working electrode, 5 mg of photocatalyst and 5 mL DMF were firstly mixed by sonication for 1 h. Then, 40 μL of the suspension was spin-coated on an F-doped SnO_2_ transparent conductive glass (FTO) slide with a specific round area (0.2826 cm^2^), and naturally dried at room temperature. Subsequently, the sample was added to 10 μL of Nafion solution (0.05%) and naturally dried at room temperature. Photoelectrochemical measurements were conducted in a three-electrode cell in an aqueous Na_2_SO_4_ electrolyte (0.2 M, pH 6.6) using a VSP-300 (Biologic, Seyssinet-Pariset, France) electrochemical analyzer. The catalyst electrode, saturated calomel electrode, and Pt plate were utilized as the working electrode, the reference electrode, and the counter electrode, respectively. The electrolyte solution was 0.1 M Na_2_SO_4_ solution. Photocurrent densities were measured under a 300 W Xenon lamp (Perfect Light PLSSXE 300, Beijing, China) with a 420 nm cutoff filter. Electrochemical impedance spectroscopy (EIS) was tested at a 5 mV sinusoidal AC perturbation over the frequency range 0.1~10^5^ Hz at −0.2 V. Linear sweep voltammetry (LSV) measurements were performed at a scan rate of 20 mV s^−1^ in the range −1.0~0.7 V. Carrier density was estimated through the Mott–Schottky relation according to Equation (4):

(4) $$\frac{1}{C_{\mathrm{sc}}{}^{2}}=\frac{2}{\varepsilon \varepsilon_{0}A^{2}eN_{d}\;}(V\;-\;V_{\mathrm{fb}}\;-\;\frac{k_{b}T}{e})$$

where *C*_sc_ is the capacitance of the space charge region, *e* is the elementary charge of an electron (1.602 × 10^−19^ C), *ε*_0_ is the permittivity of vacuum (8.854 × 10^−14^ F cm^−1^), *ε* is the dielectric constant of carbon nitride polymer (*ε* ≈ 8), *A* is the electrochemically active surface area (0.28 cm^2^), *V* is the applied voltage, *V*_fb_ is the flatband potential, and *N*_d_ is the donor density (carrier concentration). *T* is the absolute temperature, *k*_b_ is the Boltzmann constant, and *k*_b_*T*/e is about 0.026 V at room temperature.

### 3.9. Photocatalytic Hydrogen Evolution

Hydrogen evolution was measured in a Ceaulight CEL-SPH2N-D5 closed gas-circulation–evacuation system. Photocatalyst powder (50 mg) was dispersed in an aqueous solution (100 mL) containing triethanolamine (10 mL) or 0.1 mol L^−1^ ascorbic acid (H_2_A) with an adjusted pH of 3.5, and added into a Pyrex top-irradiation reaction vessel connected to a glass closed-gas system. The catalyst was stirred, loaded with 3 wt% Pt using an in situ photodeposition approach with H_2_PtCl_6_·6H_2_O, and subjected to vacuum degassing for several times to completely remove air. Photocatalytic H_2_ evolution array was conducted in a vacuum at 6 °C to avoid the evaporation of water, which interferes with light irradiation. Gas products were determined by a gas chromatograph with a 5A sieve column. The system was then irradiated under a Perfect Light PLSSXE 300 W Xe lamp (Beijing, China) equipped with an appropriate long-pass cutoff filter and maintained at room temperature by a flow of cooling water. The generated gases were detected by a Shimadzu GC-2014C gas chromatograph (Kyoto, Japan) equipped with a thermal conductive detector (5A sieve) and argon as the carrier gas.

### 3.10. Apparent Quantum Efficiency for H_2_ Evolution Measurement

The apparent quantum efficiency (AQY) was calculated according to Equation (5):

(5) $$\begin{array}{c} AQY=\frac{2\times \mathrm{number}\;\mathrm{of}\;\mathrm{evolved}\;\mathrm{hydrogen}\;\mathrm{molecules}}{\mathrm{number}\;\mathrm{of}\;\mathrm{incident}\;\mathrm{photons}}\;\times \;100\%\\ AQY=\frac{N_{e}}{N_{p}}\;\times \;100\%=\frac{2\times M\times N_{A}}{\frac{E_{total}}{E_{photon}}}\times 100\%=\frac{2\times M\times N_{A}}{\frac{S\times P\times t}{h\times \frac{c}{\lambda }}}\times 100\%\;=\frac{2\times M\times N_{A}\times h\times c}{S\times P\times t\times \;\lambda }\times 100\% \end{array}$$

where *M* is the number of H_2_ molecules (mol), *N*_A_ is the Avogadro constant (6.022 × 10^23^ mol), *h* is the Planck constant (6.626 × 10^−34^ J·s), *c* is the speed of light (3 × 10^8^ m/s), *S* is the irradiation area (cm^2^), *P* is the intensity of irradiation light (W/cm^2^), *t* is the photoreaction time (s), and *λ* is the wavelength of the monochromatic light (m).

The AQY of H_2_ generation was tested by using different band-pass filters. A PLSSXE 300 W Xe lamp with a band-pass filter (420 ± 15 nm) was used as the light source. The intensity of irradiation light was 5.369 mW/cm^2^. The H_2_ evolution amount of 5% CdSe/CN was 456.2 μmol for the 5 h reaction.


(6) $$\mathrm{AQY}=\frac{2\times 456.2\times 10^{-6}\times 6.02\times 10^{23}\times 6.626\times 10^{-34}\times 3.0\times 10^{8}}{26.42\times 5.369\times 10^{-3\;}\times 5\times 3600\times 420\times 10^{-9}}\;\times \;100\%=10.2\%$$

### 3.11. Photocatalytic CO_2_ Reduction

Catalyst powder (30 mg), 2,2′-bipyridyl (15 mg), H_2_O (1 mL), acetonitrile (3 mL), triethanolamine (1 mL), and CoCl_2_·6H_2_O (1 μmol) were mixed under stirring in a Schlenk flask (80 mL). The mixture was subjected to vacuum degassing and backfilling with pure CO_2_ gas for 3 times. The system was filled with CO_2_ (1 atm) after the last cycle. The photocatalytic CO_2_ reduction array was performed at 30 °C in an atmospheric system, free from obvious evaporation disturbance under the experimental conditions. The system was then irradiated with a Perfect Light PLSSXE 300 W Xe lamp (Beijing, China) with a 420 nm cutoff filter. The gas products were tested using a 7890B gas chromatograph (Agilent, Santa Clara, CA, USA) equipped with a methanizer, flame ionization detector, thermal conductivity detector, and TDX-1 packed column with argon as the carrier gas.

### 3.12. Computational Details

The slab calculations were performed with the Vienna ab initio package (VASP) [57,58,59]. In the calculations, within the framework of density functional theory (DFT), the PBE exchange-correlation functional [60] and the dispersion interaction corrected by the D3 scheme were considered [61]. The cutoff energy for the plane waves was 400 eV, and the atomic core region was described by PAW pseudopotentials [62]. Dipole correction along the z-direction was taken into consideration [63]. 5 × 3 × 1 and 4 × 4 × 1 k-point meshes were used for CN and CdSe (111) in DFT calculation, respectively. The vacuum layer of slab model was set to 15 Å.

The molecule-level calculations were performed using the Gaussian 09 programs [64]. The structures were fully optimized with the B3LYP [65,66] method and Ahlrichs’ split-valence def2-SVP basis set [67]. Grimmes’s DFT-D3 dispersion correction was used to describe the van der Waals interaction.

## 4. Conclusions

In summary, a zero-dimensional/one-dimensional S-scheme heterojunction of CdSe quantum dots coupled with polymeric carbon nitride nanorods were prepared via a chemical impregnation method. Five percent CdSe/CN composite showed the best photocatalytic efficiency in water splitting, with a hydrogen evolution rate of 20.1 mmol g^−1^ h^−1^ and apparent quantum yield of 10.2% at 420 nm irradiation, and exhibited a CO production rate of 0.77 mmol g^−1^ h^−1^ in CO_2_ reduction. The superior reactivity of CdSe/CN heterojunction displays toward water splitting and carbon dioxide photoreduction are mainly attributed to the more efficient charge-carrier separation rate, stronger light absorption ability, and more abundant active sites. The higher work function value of polymeric carbon nitride than CdSe leads to the transfer of electrons from CdSe quantum dots to polymeric carbon nitride nanorods upon hybridization, and interfacial electric field is created at heterointerfaces. The photoinduced electrons in the conduction band of the polymeric carbon nitride nanorods then immigrate to the valence band of CdSe, confirming an S-path of charge transfer. This work demonstrates the possibility of employing zero-dimensional/one-dimensional S-scheme heterojunction photocatalysts for solar energy conversion.

## Data Availability

Not applicable.

## Supplementary Materials

The following supporting information can be downloaded at: https://www.mdpi.com/article/10.3390/molecules27196286/s1, Figures S1–S14 and Tables S1–S5, with references [39,68,69,70,71,72,73,74,75,76]. Figure S1: SEM images of CN nanorods; Figure S2: SEM images of 5% CdSe/CN; Figure S3: (a) TEM image, (b) HRTEM image, (c) SAED image, and (d) EDX image of CdSe QD solutions; Figure S4: TEM-EDX image of 5% CdSe/CN hybrid; Figure S5: XPS survey spectra of CN, CdSe and 5% CdSe/CN hybrid; Figure S6: Tauc plots of (a) CN nanorods, CdSe/CN hybrids, and (b) CdSe QDs; Figure S7: (a) Amount of hydrogen evolved from 5% CdSe/CN using ascorbic acid (H_2_A) as sacrificial reagent by changing pH value. (b) AQY of 5% CdSe/CN for photocatalytic H_2_ evolution with different photocatalyst weight.; Figure S8: (a) XRD pattern, (b) FTIR spectra, and (c) UV–Raman spectra of 5% CdSe/CN before and after the hydrogen evolution reaction; Figure S9: SEM images of 5% CdSe/CN after photocatalytic reactions; Figure S10: (a) XRD pattern, (b) FTIR spectra, and (c) UV–Raman spectra of 5% CdSe/CN before and after the CO_2_ conversion reaction; Figure S11: Mott–Schottky plots of (a) CN nanorods, (b) 5% CdSe/CN, and (c) CdSe QDs; Figure S12. Mott–Schottky plots of CN nanorods and 5% CdSe/CN to calculate the charge-carrier density; Figure S13. Linear sweep voltamentary for CN and 5% CdSe/CN; Figure S14. EPR spectra of CN and 5% CdSe/CN; Table S1: Fitted fluorescence decay components of CN nanorods and CdSe/CN (λ = 375 nm); Table S2. Literature values of AQY for CN-based photocatalysts in hydrogen evolution; Table S3: Various controlled experiments for CO_2_ reduction; Table S4: The reactivity of photocatalysts for CO_2_ reduction. Table S5. Calculated values of equivalent circuit elements for CN and 5% CdSe/CN samples.

## Sample Availability

Samples of the compounds of CdSe/CN hybrids are available from the authors.
