# Supplementary material for: Designing a 0D/1D S-Scheme Heterojunction of Cadmium Selenide and Polymeric Carbon Nitride for Photocatalytic Water Splitting and Carbon Dioxide Reduction
Source: Molecules. 2022 Sep 23;27(19):6286. doi: 10.3390/molecules27196286 (PMC9572265; doi:10.3390/molecules27196286)
Supplement: Supplementary file 1 [file molecules-27-06286-s001.zip › molecules-1909441-supplementary.pdf]

Supplementary Materials

Designing a 0D/1D S-Scheme Heterojunction of Cadmium Selenide and Polymeric Carbon Nitride for Photocatalytic Water Splitting and Carbon Dioxide Reduction

Yayun Wang [1], Haotian Wang [1], Yuke Li [2], Mingwen Zhang [3,*] and Yun Zheng [1,*]

1 Xiamen Key Laboratory of Optoelectronic Materials and Advanced Manufacturing, College of Materials Science and Engineering, Huaqiao University, Xiamen 361021, China
2 Department of Chemistry and Centre for Scientific Modeling and Computation, Chinese University of Hong Kong, Shatin, Hong Kong, China
3 Fujian Provincial Key Lab of Coastal Basin Environment, School of Materials and Environment Engineering, Fujian Polytechnic Normal University, Fuqing 350300, China
* Correspondence: mwzhang1989@163.com (M.Z.); zheng-yun@hqu.edu.cn (Y.Z.)

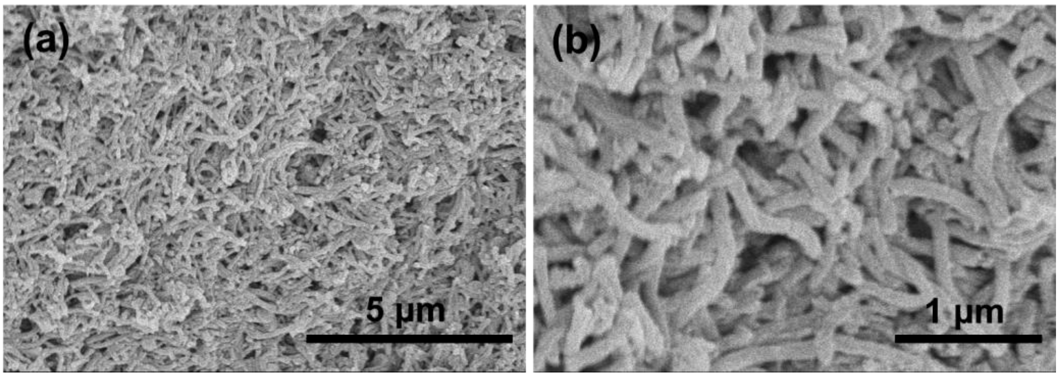

Figure S1. SEM images of CN nanorods.

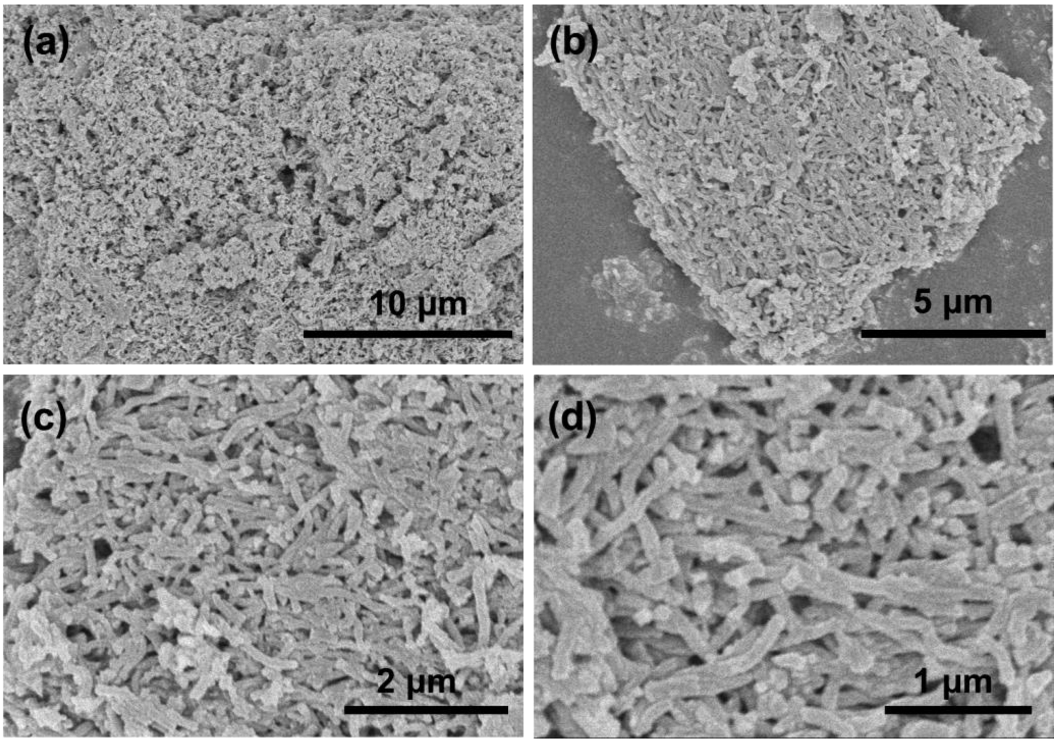

Figure S2. SEM images of 5% CdSe/CN hybrid.

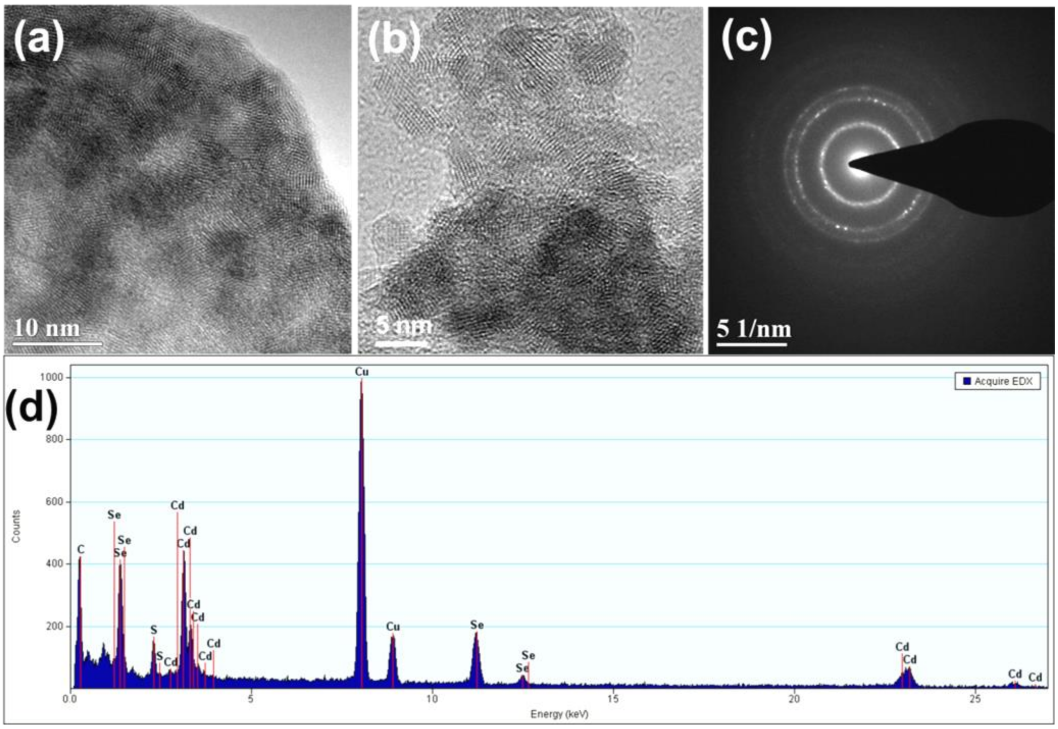

Figure S3. (a) TEM image, (b) HRTEM image, (c) SAED image, and (d) EDX image of CdSe QD solutions.

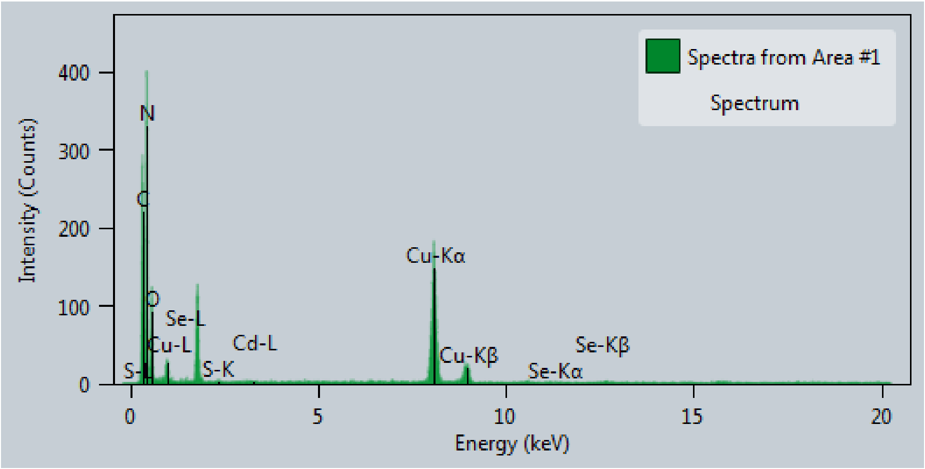

Figure S4. TEM-EDX images of 5% CdSe/CN hybrid.

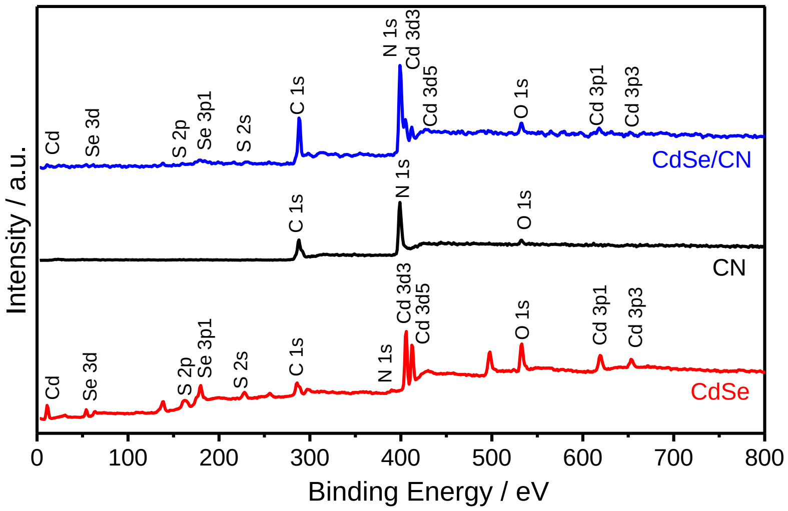

Figure S5. XPS survey spectra of CN, CdSe and 5% CdSe/CN hybrid.

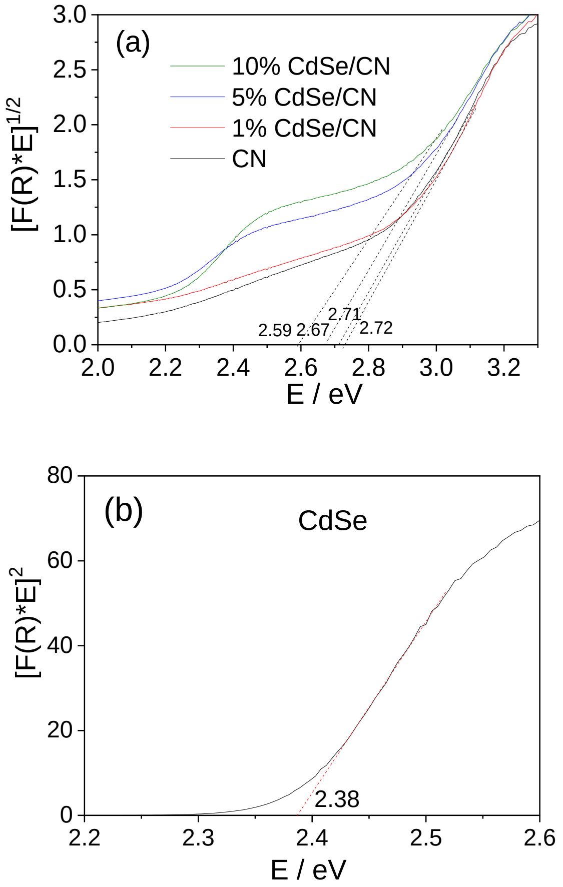

Figure S6. Tauc plots of (a) CN nanorods, CdSe/CN hybrids and (b) CdSe QDs.

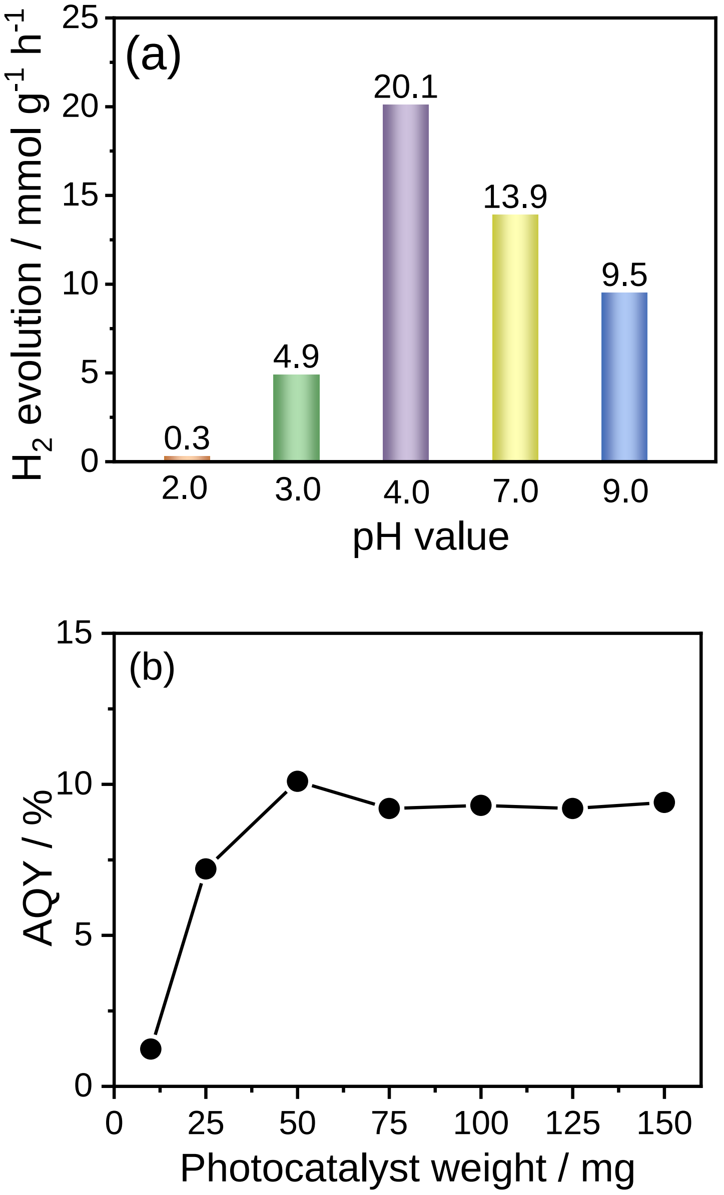

Figure S7. (**a**) Amount of hydrogen evolved from 5% CdSe/CN using ascorbic acid (H_2A) as sacrificial reagent by changing pH value. (**b**) AQY of 5% CdSe/CN for photocatalytic H_2 evolution with different photocatalyst weight.

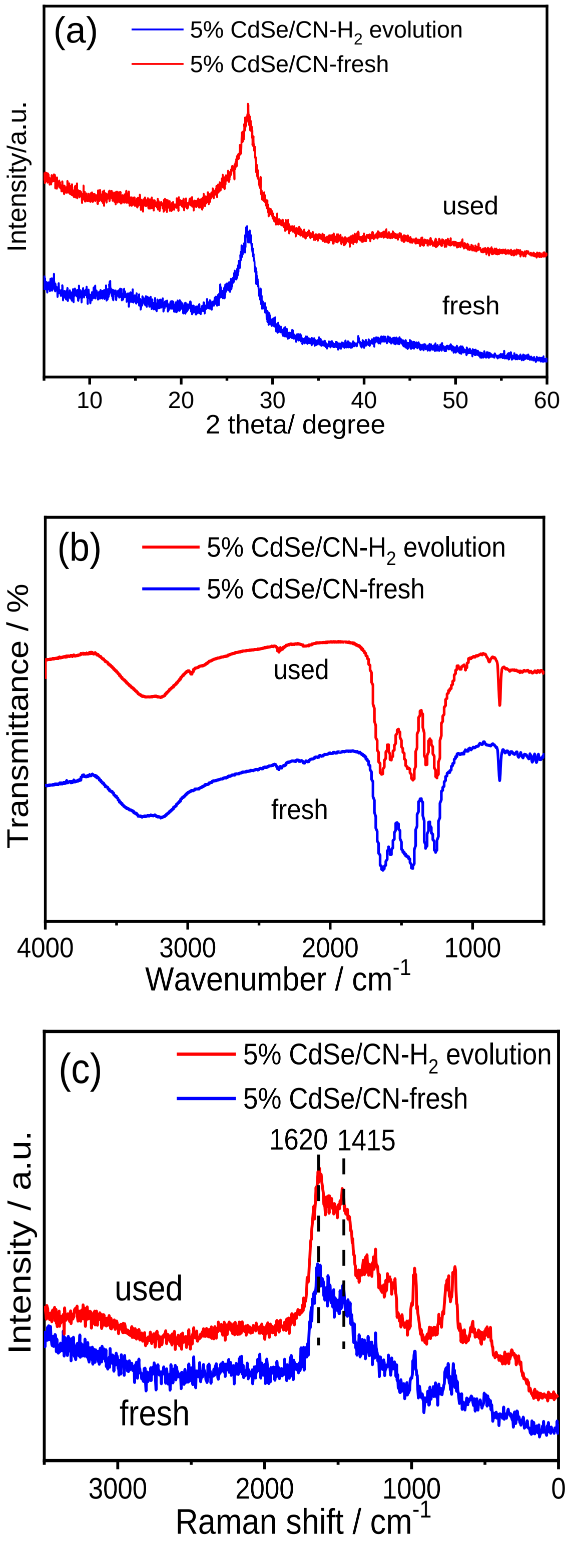

Figure S8. (a) XRD pattern, (b) FT-IR spectra and (c) UV-Raman spectra of 5% CdSe/CN before and after the hydrogen evolution reaction.

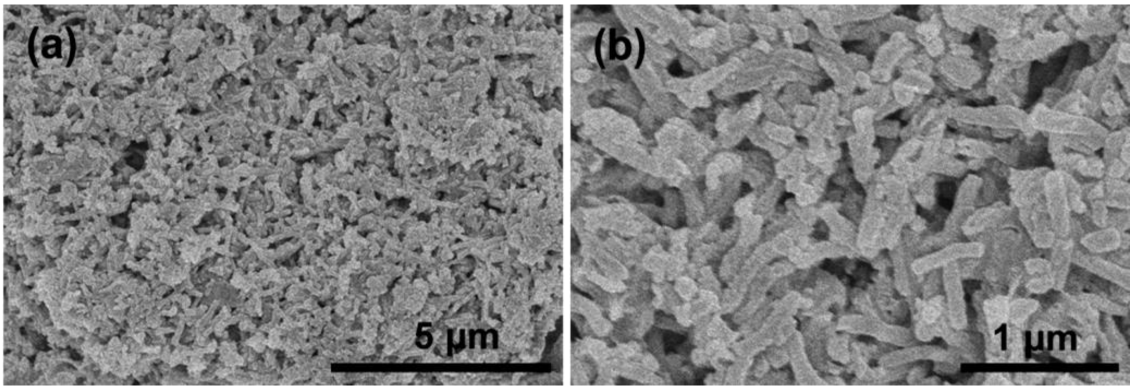

Figure S9. SEM images of 5% CdSe/CN after photocatalytic reactions.

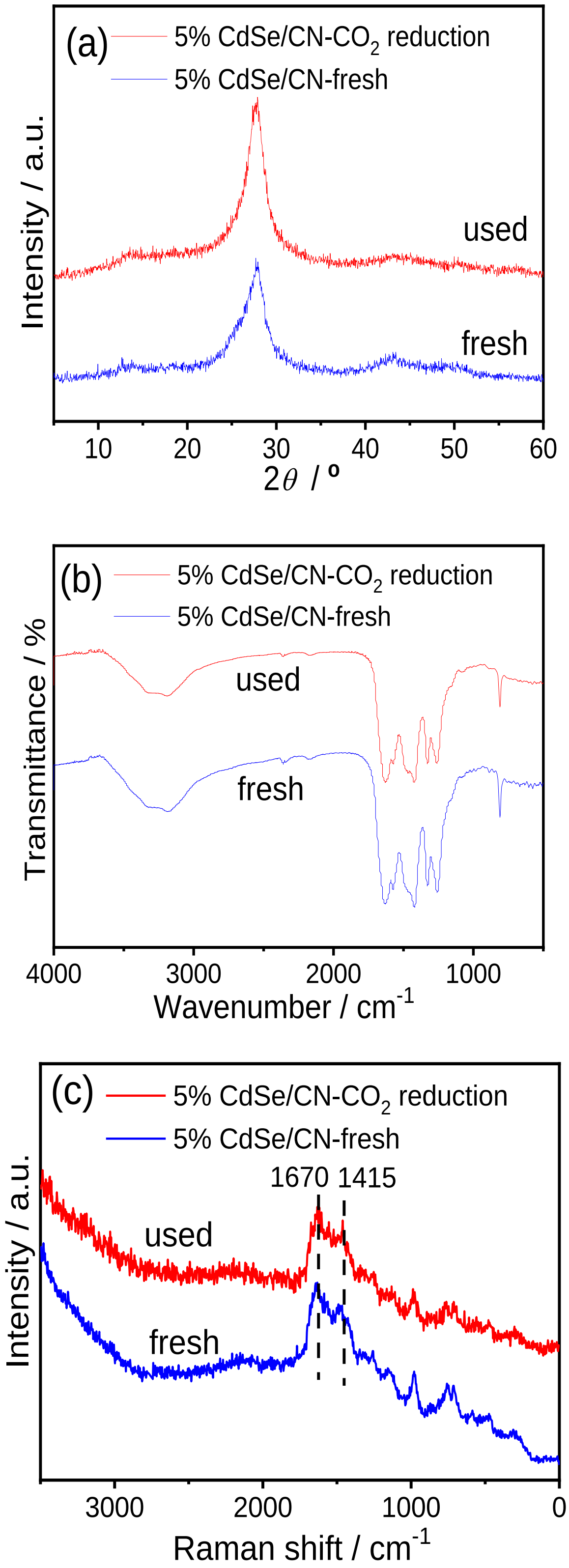

Figure S10. (a) XRD pattern, (b) FT-IR spectra, and (c) UV-Raman spectra of 5% CdSe/CN before and after the CO_2 conversion reaction.

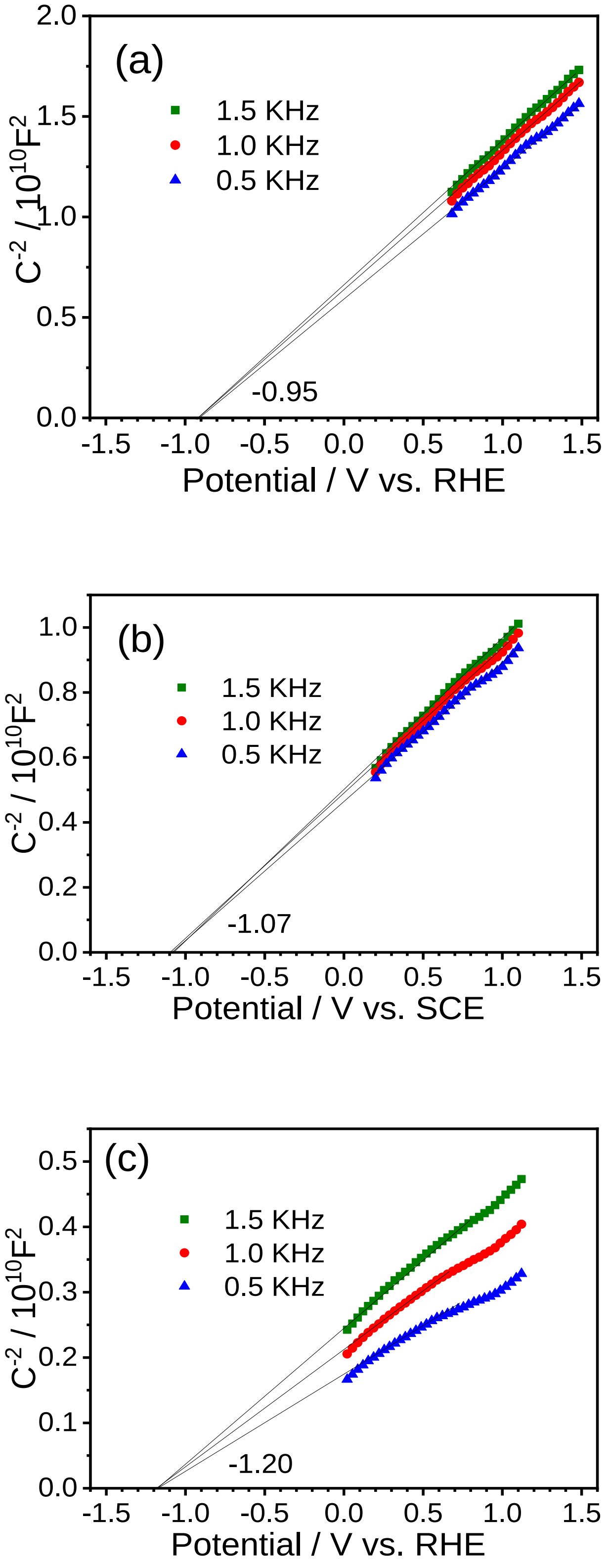

Figure S11. Mott-Schottky plots of (a) CN nanorods, (b) 5% CdSe/CN and (c) CdSe QDs.

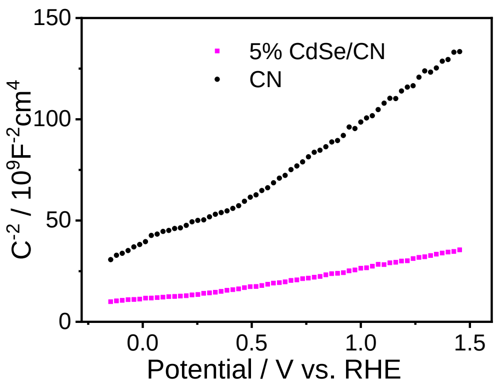

Figure S12. Mott-Schottky plots of CN nanorods and 5% CdSe/CN to calculate the charge carrier density.

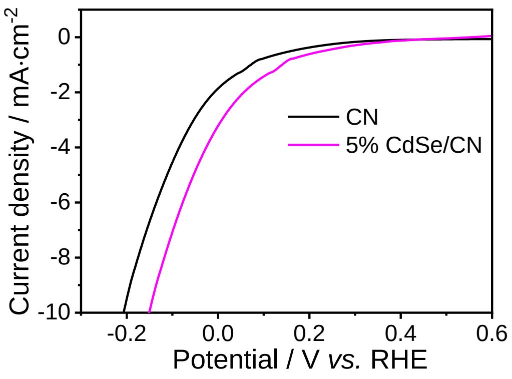

Figure S13. Linear sweep voltamentary for CN and 5% CdSe/CN.

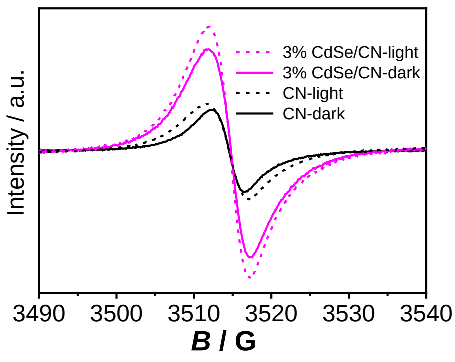

Figure S14. EPR spectra of CN and 5% CdSe/CN.

Table S1. Fitted fluorescence decay components of CN nanorods and 5% CdSe/CN (λ = 375 nm).

Sample	τ_1 (ns)	τ_2 (ns)	$\tau_{average}$ (ns)	x^2
CN nanorods	1.15 (86.3%)	9.71 (13.7%)	2.32	0.986
5% CdSe/CN	1.03 (76.9%)	5.83 (23.1%)	2.14	0.993

short lifetime (τ_1), long lifetime (τ_2) and average lifetime ($\tau_{average}$).

Table S2. Literature values of AQY for CN-based photocatalysts in hydrogen evolution.

Photocatalyst	Photocatalyst loading (mg)	Co-catalysts	Incident wavelength;λ_i (nm)	AQY (%)	Reference
CdSe/CN	20	3 wt % Pt	420 nm	10.2	This work
18 wt% CdSe-TF-g-C_3N_4 sheet	5	/	460 nm	13.9	[39]
Porous g-C_3N_4 sheet	50	3 wt % Pt	420 nm	1.5	[68]
			580nm	6.3	
			680nm	4.4	
Carbon QDs on g-C_3N_4 nano tubes	50	3 wt % Pt	420 nm	10.9	[69]
P-doped g-C_3N_4 nanoflakes	10	3 wt % Pt	420 nm	6.7	[70]
			600nm	0.2	
Crystalline g-C_3N_4 nanosheet	50	3 wt % Pt	420 nm	9.0	[71]
Thin, porous g-C_3N_4 sheet	50	3 wt% Pt	425 nm	6.8	[72]
Defect-rich amorphous carbon nitride (DACN)	50	3 wt% Pt	400 nm	34.4	[73]
			425nm	31.9	
CdS QDs on WS_2/g-C_3N_4 nanosheets	10	/	400 nm	5.4	[74]
Few-layered polymeric CN	50	3 wt% Pt	405 nm	11.3	[75]
C_3N_4 sheet/MnO_2	100	/	420 nm	3.8	[76]

Table S3. Various controlled experiments for CO_2 reduction.

Entry	Reaction Conditions	CO [μmol]	H_2 [μmol]	CO+H_2 [μmol]
1	(a)	7.0	1.1	8.1
2	Without catalyst	n.d. (b)	n.d.	-
3	In dark	n.d.	n.d.	-
4	Using Ar instead of CO_2	n.d.	0.5	0.5
5	Without triethanolamine	n.d.	n.d.	-
6	Without bipyridine	n.d.	<0.1	<0.1
7	Without $CoCl_2·6H_2O$	n.d.	<0.1	<0.1

(a) Reaction conditions: 5% CdSe/CN catalyst (20 mg), 2,2'-bipyridyl (15 mg), H_2O (1 mL), acetonitrile (3 mL), triethanolamine (1 mL), $CoCl_2·6H_2O$ (1 μmol), CO_2 (1 bar), 40 °C, 1 h, and λ> 420 nm; (b) Not detectable.

Table S4. The reactivity of photocatalysts for CO_2 reduction.

Entry	Catalyst	CO [μmol]	H_2 [μmol]	CO+H_2 [μmol]	Turn over number (a)	Selectivity (b)/ %
1	CN nanorods	8.9	0.3	9.2	9.2	96.7
2	CdSe	0.5	0.1	0.6	0.6	83.3
3	1% CdSe/CN	15.2	0.4	15.6	15.6	97.4
4	3% CdSe/CN	17.8	0.4	18.2	18.2	97.8
5	5% CdSe/CN	23.2	0.5	23.7	23.7	97.9
6	7% CdSe/CN	20.8	0.6	21.4	21.4	97.2
7	10% CdSe/CN	18.5	0.6	19.1	19.1	96.9

Reaction conditions: catalyst (30 mg), 2,2'-bipyridyl (15 mg), H_2O (1 mL), acetonitrile (3 mL), triethanolamine (1 mL), $CoCl_2·6H_2O$ (1 μmol), CO_2 (1 bar), 30 °C, 1 h, and λ > 420 nm. (a) Turn over number = (mol amount of CO and H_2)/(mol amount of cobalt ion); (b) Selectivity =$n_{CO}/n_{(CO+H2)}$× 100; (c) Not detectable.

Table S5. Calculated values of equivalent circuit elements for CN and 5% CdSe/CN samples.

Samples	R_1 [Ω]	R_2 [Ω]	C_2 [F]
CN	145.8	$1.09×10^6$	$1.52×10^{-6}$
5% CdSe/CN	124.2	$9.73×10^5$	$3.57×10^{-6}$
